# Omics-Based Sperm-Retrieval Prediction in Non-Obstructive Azoospermia: A Critical Narrative Review and Validation Framework

**DOI:** 10.3390/genes17091088

**Published:** 2026-09-10

**Authors:** Aris Kaltsas, Maria-Anna Kyrgiafini, Eleftheria Markou, Michael Chrisofos

**Affiliations:** 1 Third Department of Urology, Attikon University Hospital, School of Medicine, National and Kapodistrian University of Athens, 12462 Athens, Greece; ares-kaltsas@hotmail.com; 2 Laboratory of Genetics, Comparative and Evolutionary Biology, Department of Biochemistry and Biotechnology, University of Thessaly, Viopolis, Mezourlo, 41500 Larissa, Greece; mkyrgiafini@uth.gr; 3 Department of Microbiology, University Hospital of Ioannina, 45500 Ioannina, Greece; eleftheria.markou@uoi.gr

**Keywords:** non-obstructive azoospermia, sperm retrieval, micro-TESE, omics, biomarker, prediction model, external validation, calibration, clinical utility

## Abstract

In non-obstructive azoospermia (NOA), microdissection testicular sperm extraction can provide sperm for intracytoplasmic sperm injection, but retrieval fails in approximately half of procedures. Genomic, transcriptomic, noncoding RNA, proteomic, metabolomic, and microbiome studies have reported molecular associations and prediction estimates. This critical narrative review examines the requirements for an assay–model system to support preoperative retrieval counseling. A focused PubMed/MEDLINE search updated on 31 August 2026 and targeted reference checking identified representative human reports and methodological guidance. Selected reports mainly illustrate discovery, development, and same-source evaluation. Common limitations include small cohorts, local assay optimization, heterogeneous outcomes, incomplete calibration, and uncertain transportability. Established karyotyping and Y-chromosome testing must be distinguished from discovery-scale genomics, which currently supports etiologic and qualified genotype-specific counseling rather than a universal calibrated retrieval model. A routine-variable multicenter model reported an external-cohort area under the receiver-operating-characteristic curve (AUC) of 0.8301, although cohort provenance, calibration, and clinical utility require independent confirmation. An author-developed seven-gate framework integrates clinical-question definition, assay specification, model development, internal validation, external evaluation, incremental value, and prospective impact. Future omics studies should test incremental value beyond a prespecified routine-variable model in the same patients and assess calibration, threshold consequences, net benefit, assay failure, cost, and patient outcomes.

## 1. Introduction

Non-obstructive azoospermia (NOA) is a severe form of male-factor infertility in which spermatogenesis is absent or too limited for sperm to appear in the ejaculate. Surgical retrieval may obtain autologous sperm for intracytoplasmic sperm injection (ICSI), but retrieval and live birth are distinct outcomes. Current European Association of Urology guidance recommends microdissection testicular sperm extraction (micro-TESE) for men with NOA, although the recommendation is weak [1]. Across 117 heterogeneous testicular sperm extraction (TESE) series including 21,404 men, the pooled retrieval rate was approximately 47% per procedure [2]. This population average does not estimate an individual probability. Unsuccessful retrieval remains common, and a false-negative prediction could deny the only opportunity to attempt genetic fatherhood with autologous sperm.

The relevant question is whether information available at a specified decision point can provide a trustworthy patient-level probability for a specified procedure. A group-level association with spermatogenesis does not answer that question. Such a probability could support counseling, consent, referral, and procedure planning. It should not deny an attempted retrieval unless prospective evaluation shows patient benefit and an acceptably low rate of false-negative exclusion.

**Figure 1:**
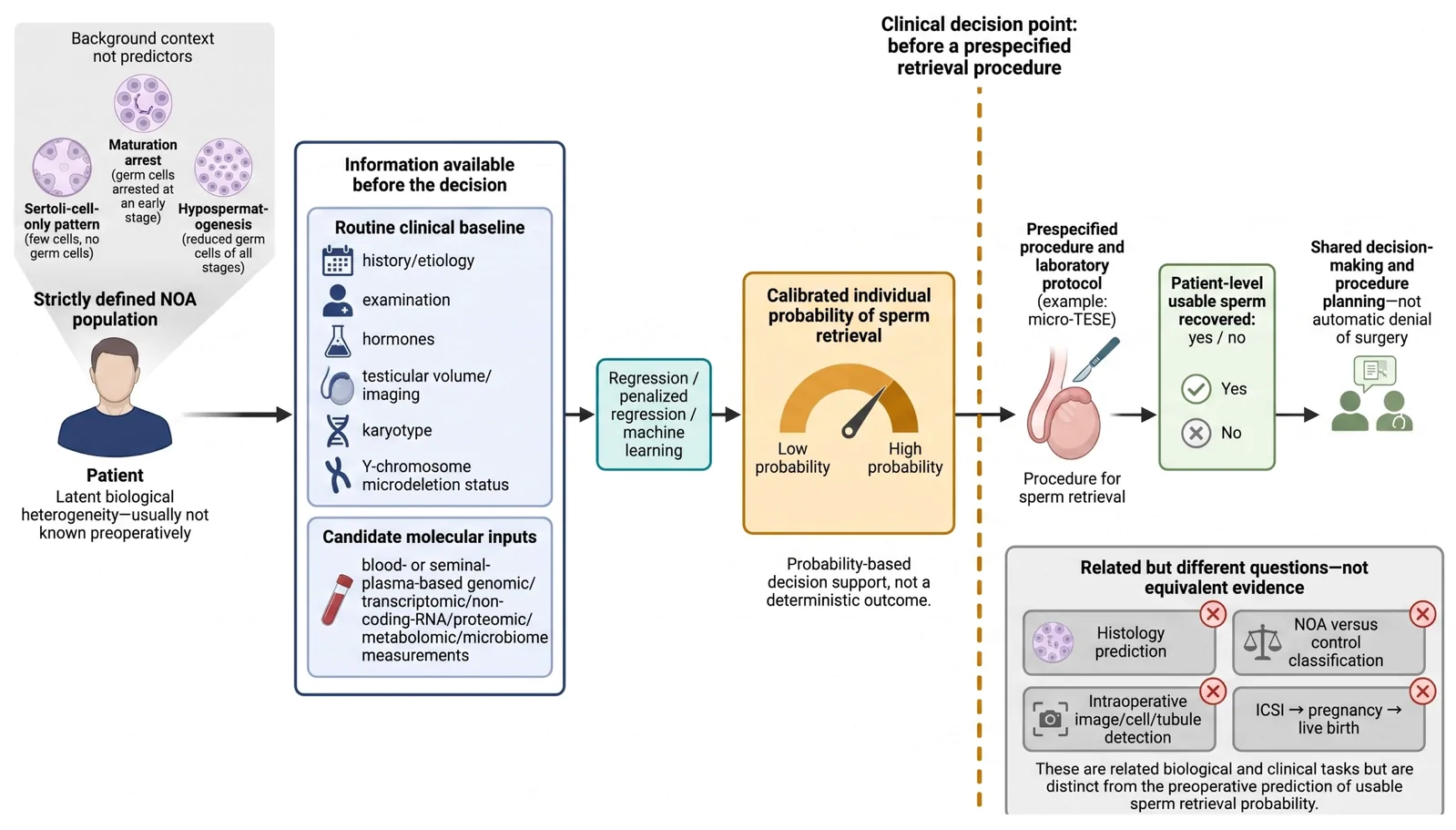
The preoperative prediction task in non-obstructive azoospermia (NOA). (**Left**) The intended-use population. Underlying histology is usually unknown before surgery and is shown as background context, not as a predictor. (**Center**) Routine clinical, hormonal, and genetic data are combined with candidate molecular inputs to estimate a calibrated individual probability. (**Right**) The prespecified retrieval procedure, patient-level outcome, and shared decision-making supported by that probability. The gray box lists related tasks, including histology prediction, NOA-versus-control classification, intraoperative detection, and outcomes after intracytoplasmic sperm injection (ICSI). These tasks are not equivalent evidence for preoperative retrieval prediction. Created in BioRender. Kaltsas, A. (2026) https://BioRender.com/vq1d0s4, accessed on 14 August 2026. Arrows indicate the conceptual workflow from preoperative information through probability estimation to the specified retrieval procedure, outcome assessment, and clinical decision support; they do not imply causal relationships.

The information available at that decision point is largely routine. Age, etiology, testicular volume, serum follicle-stimulating hormone (FSH), luteinizing hormone, testosterone, and karyotype are assessed before surgery. Individual clinical markers have limited predictive value. In a 21-study meta-analysis, FSH and testicular volume showed weak discrimination [3]. A later 34-report synthesis found no significant pooled prediction by FSH or inhibin B and substantial heterogeneity for anti-Müllerian hormone [4]. No combination is a definitive general predictor across unselected NOA [5]. A correctly delimited complete AZFa or AZFb deletion is an important exception. Sperm retrieval is considered virtually impossible, and testing should follow dedicated EAA/EMQN guidance [6]. Histology can be strongly informative when already known. However, a separate predictive biopsy is invasive, and a small specimen may miss focal spermatogenesis. In a retrospective series of 50 men undergoing micro-TESE, sperm appeared in 11 concurrent diagnostic specimens and in 22 larger processed therapeutic specimens [7]. This same-procedure comparison illustrates sampling and processing limitations, not prospective validation of a preoperative biopsy model. A new biomarker baseline must therefore contain only variables available at the intended prediction time. Figure 1 defines the prediction task and separates it from related questions.

Molecular measurement may extend that baseline. Seminal plasma, serum, and testicular tissue contain nucleic acids, extracellular vesicles (EVs), proteins, metabolites, and microbial or host–microbial signals. These signals may reflect spermatogenic activity, and reviews have described many biologically plausible candidates [8,9]. High-dimensional assays can capture distributed signals that a single hormone cannot. Regression and machine-learning methods can combine molecular and routine clinical information. Machine learning is a modeling method, not an omics modality. A complex algorithm does not reduce the evidential requirements [10].

Recent reviews address related but distinct questions. Biomarker reviews describe candidate molecular classes and their biological rationale, including an earlier review of seminal plasma as a liquid biopsy [8,9]. Jamalirad et al. systematically reviewed machine-learning models for micro-TESE and assessed risk of bias with the Prediction model Risk Of Bias ASsessment Tool (PROBAST) [11]. The present review focuses on the combined evaluation of a molecular assay and its prediction model. This approach integrates analytical validity, model development, external evaluation, incremental value, and clinical impact. It also distinguishes reports, cohorts, model specifications, and dataset-specific evaluations.

## 2. Review Design and Scope

### 2.1. Review Question and Design

This article is a critical narrative review informed by a focused PubMed/MEDLINE search and reference checking. It asks which analytical and prediction-model requirements should be satisfied before an omics assay can support preoperative retrieval counseling in NOA. Selected reports illustrate methodological issues across discovery, assay verification, model development, internal validation, external evaluation, and clinical impact. The review does not estimate the prevalence of deficiencies or establish a field-wide absence of qualifying models. The synthesis was prepared with reference to the Scale for the Assessment of Narrative Review Articles (SANRA) [12].

### 2.2. Literature Identification and Selection

On 31 August 2026, a focused PubMed/MEDLINE literature-mapping search was rerun for records published from 1 January 2010 through 31 August 2026. The reproducible query combined terms for NOA, sperm retrieval, molecular biomarkers or omics, and prediction or validation. It returned 221 PubMed records before eligibility assessment. No language, human-study, or document-type filter was applied at the query stage. Reference lists of relevant primary reports, reviews, clinical guidance, and methodological publications were also checked.

Titles of all 221 records were inspected. An abstract-level audit covered 22 reports, comprising directly molecular or potentially omitted records and tabulated reports requiring source re-checking. This was a single-reviewer narrative mapping exercise, not a duplicated systematic screen. Supplementary Table S1 provides the complete strategy, database platform, dates, restrictions, record counts, table rules, and source-access procedures. Supplementary Table S2 lists potentially relevant reports not included in the main tables and gives one reason for each decision.

### 2.3. Scope and Units of Analysis

Human studies of patient-level sperm retrieval in men with NOA received priority. Mixed obstructive and non-obstructive populations, histology prediction, NOA-versus-control classification, and intraoperative detection were cited only when they clarified a methodological issue. Such reports were not treated as direct evidence for preoperative retrieval prediction. First-attempt and salvage procedures were considered separately [13]. Fine-needle aspiration (FNA), testicular sperm aspiration (TESA), conventional TESE, and micro-TESE were also distinguished.

Selection for the main tables was purposive. Reports were chosen to illustrate distinct molecular modalities, intended uses, and evaluation designs rather than to create an exhaustive inventory. Terminology was informed by the CHecklist for critical Appraisal and data extraction for systematic Reviews of prediction Modeling Studies (CHARMS) [14]. It also followed the Transparent Reporting of a multivariable prediction model for Individual Prognosis Or Diagnosis (TRIPOD) statement and its artificial-intelligence extension (TRIPOD+AI) [15,16]. Appraisal vocabulary drew on PROBAST, its artificial-intelligence extension (PROBAST+AI), and the TRIPOD extension for systematic reviews and meta-analyses (TRIPOD-SRMA) [17,18,19]. A report, study, cohort, specified model, and dataset-specific evaluation were treated as separate units. Reports from overlapping cohorts were not treated as independent replication. No formal systematic-review risk-of-bias rating or prevalence estimate was performed.

### 2.4. Descriptive Appraisal of Selected Reports

Selected reports were examined for population, prediction time, procedure, outcome, specimen, assay, sample size, missing data, model specification, internal validation, calibration, external evaluation, incremental value, clinical utility, and reproducibility. Full texts and Supplementary Files were sought for revised claims and tabulated reports. When only an abstract or incomplete source was accessible, only explicitly reported information was extracted. Remaining items were recorded as not verified from the accessible source and were not interpreted as absent from the full publication. Supplementary Table S3 records the access status of the reports central to revised claims.

When one area under the receiver-operating-characteristic curve (AUC) was tabulated, the estimate came from the evaluation dataset with the greatest separation from model development. The order was independent-center evaluation, same-center temporal evaluation, blinded same-setting holdout, random same-cohort holdout, and development-only or case–control analysis. Within that dataset, the primary or recommended model was used instead of the largest reported AUC.

## 3. Omics Signals: Discovery and Validation Maturity

### 3.1. What Counts as an Omics Prediction Signature?

An association between a molecular feature and retrieval is not yet a prediction model, and a model tested in a random split from the same source is not independently validated. Clinical maturity lies on a continuum: biological discovery, assay verification, model development, internal validation, temporal evaluation, center-held-out geographic evaluation and prospective impact assessment. A differential-expression *p*-value, a single-marker receiver-operating-characteristic curve or an apparently high development AUC may justify further study; none alone establishes that predicted probabilities will be accurate or useful in another laboratory.

Predictor modality and modeling method are orthogonal. Transcript abundance, proteins, metabolites, or microbial features may be analyzed with conventional regression, penalized regression, tree-based learning, or neural networks. The same algorithms may use only clinical or imaging variables. Routine karyotyping and established Y-chromosome testing should be separated from discovery-scale genomics and epigenomics. The former are available clinical baseline variables. The latter generate high-dimensional candidate information with additional ancestry, multiple-testing, and transportability concerns [20,21].

An omics signature may also encode differences in cell composition rather than a stable, transportable molecular mechanism. Severe germ-cell depletion can alter tissue, seminal-plasma and extracellular-vesicle profiles and produce excellent local separation between retrieval-positive and retrieval-negative groups. Such a signature may largely proxy histology and weaken when phenotype distribution, specimen handling or referral case mix changes. Primary single-cell evidence supports this concern: Zhao et al. profiled more than 80,000 testicular cells from ten healthy donors and seven men with NOA and demonstrated etiology-specific differences in Sertoli-cell states [22]. Single-cell atlases and computational deconvolution may identify the cellular source of a bulk signal, but they cannot substitute for patient-level evaluation of a prespecified assay–model system in a new center.

Clinical transportability must be established for the complete assay–model system. A molecular feature becomes clinically evaluable only after the intended population, procedure, specimen workflow, analytical method, preprocessing, model specification, calibrated output, and action threshold are defined. Evaluation must then address both laboratory reproducibility and model performance in new centers.

### 3.2. Transcriptomic and Noncoding RNA Approaches

Seminal RNA is appealing because it may provide a noninvasive readout of testicular and reproductive-tract biology. Barceló et al. reported that seminal extracellular-vesicle miR-31-5p distinguished secretory from obstructive azoospermia with 92.9% sensitivity and 80.0% specificity. In a separate exploratory analysis of 12 men with severe spermatogenic failure—eight with and four without intratesticular sperm—a model combining miR-539-5p, miR-941 and FSH perfectly classified the development sample, without independent validation [23]. Extracellular RNA is evidently informative here; separating secretory from obstructive azoospermia, however, is a different task from telling an individual man whether sperm will be found.

Xie et al. derived a nine-EV-lncRNA panel in 96 men with NOA sampled across three centers. The 30-patient training and 66-patient validation groups were created by random allocation, with reported AUCs of 0.986 and 0.960 [24]. The results are promising, but multicenter recruitment does not by itself constitute geographic validation. Because centers were not held out, the design remains an internal random holdout of pooled data and does not test whether a prespecified assay and model transport to a new laboratory.

Ji et al. used testicular tissue from six men for circular RNA (circRNA) discovery and measured selected circRNAs in preoperative seminal plasma from 52 men with idiopathic NOA. Three circRNAs had individual AUCs of 0.891–0.928 and a least absolute shrinkage and selection operator (LASSO)-combined model had an AUC of 0.958 [25]. Zhang et al. screened seminal-plasma microRNAs (miRNAs), fitted an ordered logistic model in 56 specimens and evaluated it in a blinded same-setting test set of 40 specimens; the binary retrieval AUC was 0.927 [26]. The latter design is stronger than reporting performance in the fitting data, but the test set included fertile controls within a three-level framework and did not test inter-center assay transfer.

Other small-RNA and messenger RNA (mRNA) studies reinforce both the biological signal and the limited validation. Chen et al. selected an extracellular-vesicle PIWI-interacting RNA model after sequencing eight NOA and eight control samples [27]. AUCs were 0.82 and 0.83 in 20-patient training and 25-patient validation cohorts. Calibration was assessed with the Hosmer–Lemeshow test. Dai et al. evaluated cell-free seminal *BOLL* mRNA in 81 men with NOA [28]. AUC decreased from 0.846 in the random training subset to 0.752 in the validation subset, where specificity was 50%. Han et al. reported an AUC of 0.89 for seminal EV tRF-Val-AAC-010 among 41 men with NOA [29]. The two-stage case–control design did not include independent-center evaluation.

Kyrgiafini et al. performed exploratory small-RNA sequencing on testicular tissue from 40 men with azoospermia or cryptozoospermia, arranged into five sperm-presence or pregnancy-outcome groups and analyzed as two four-person RNA pools per group [30]. Differential miRNA patterns were identified, but no patient-level prespecified prediction model was developed or independently evaluated. The surgical specimen, mixed phenotype, outcome-defined groups and pooling therefore place this study at the biological-discovery stage.

Circulating extracellular-vesicle tRNA-derived fragments offer a blood-based alternative. Zhang et al. reported AUCs of 0.954 for tRF-Glu-CTC-005 and 0.921 for tRF-Gly-GCC-002 in a cohort comprising 30 men with NOA and 12 controls, and constructed a nomogram [31]. With a small case–control sample and no independent evaluation cohort, this is model-development evidence; the nomogram has not yet been tested anywhere else.

Three reports published in 2026 by Babakhanzadeh and colleagues described circRNA-based ceRNA axes in NOA [32,33,34]. Reported retrieval AUCs ranged from 0.920 to 0.983. Each estimate separates men with successful retrieval from men with failed retrieval within NOA; none separates NOA from fertile controls, and the 40 fertile men did not enter the reported discrimination analyses. All three reports describe 60 men with NOA and 40 fertile men recruited at a single center in Yazd. They report the same histological subgroups of 20 each and the same retrieval split of 29 successful and 31 failed procedures. The two reports that state an ethics approval number give the same number. These are therefore analyte panels measured in what appears to be one cohort of 100 men, and they cannot be counted as independent replication of one another.

A fourth report published on 20 August 2026 described two circulating ceRNA axes with AUCs above 0.90 [35]. Its abstract did not report the sample size or evaluation design. Potential overlap with the earlier Yazd cohort therefore requires full-text verification.

Serum and tissue studies answer related but different questions. Lv et al. fitted a six-circRNA serum risk score in a 180-patient cohort [36]. The reported AUC was 0.977, with 91.7% sensitivity and 86.5% specificity. A clearly separate external cohort was not verified. Shi et al. split 114 men with idiopathic NOA into same-center development and validation samples for a circ_MGLL nomogram [37]. AUCs were 0.868 and 0.811. The approach used testicular tissue and incorporated pathology. It is therefore invasive and procedure-informed rather than a preoperative liquid biopsy.

### 3.3. Proteomic, Metabolomic and Microbiome-Related Evidence

Protein and metabolite studies remain closer to discovery than implementation. The TEX101 enzyme-linked immunosorbent assay program included a large overall assay dataset [38]. However, the retrieval analysis included only 26 men with NOA and used an endpoint that combined spermatozoa and spermatids. TEX101 yielded an AUC of 0.69 (95% confidence interval 0.48–0.89). The overall assay count must not be presented as the retrieval-validation denominator. Earlier comparative proteomics in 40 men identified seminal LGALS3BP as a candidate associated with successful TESE [39]. Later label-free liquid chromatography–tandem mass spectrometry identified HSPA2 and LDHC in healthy, obstructive, and NOA groups [40]. Each phenotype group included only seven or eight participants. These signals require further study, but none represents a prespecified assay evaluated in another laboratory.

Gilany et al. applied gas-chromatography–mass-spectrometry profiling and quadratic discriminant analysis to seminal plasma from 11 TESE-negative men, nine TESE-positive men and ten fertile controls. Reported ROC areas exceeded 0.88, but the sample was an exploratory case–control dataset without independent validation [41]. A high-dimensional chromatographic fingerprint can generate hypotheses long before it becomes an assay with a stable cutoff and reproducible calibration from one center to the next.

Cheng et al. enrolled 35 men with cryptorchidism-associated azoospermia and 40 fertile controls; the retrieval analysis comprised 21 men who underwent micro-TESE—11 retrieval-positive and 10 retrieval-negative [42]. Metabolomic profiles separated the two retrieval subgroups when routine clinical measures did not, but the small, etiology-specific analysis and high-dimensional feature space make independent replication essential. The result should not be generalized to unselected NOA or represented as a validated universal metabolomic predictor.

Spectroscopic molecular phenotyping is adjacent to, but not interchangeable with, metabolomics. A retrospective case–control study applied seminal-plasma Raman spectroscopy and principal component analysis in 20 men with NOA [43]. Training and test estimates generated within repeated five-fold cross-validation had AUCs of 0.863 and 0.810. The sample included ten retrieval-positive and ten retrieval-negative participants. Without independent laboratory evaluation, the evidence remains proof of concept.

Microbiome evidence remains exploratory. Yao et al. combined a small human cohort with mechanistic mouse experiments [44]. The reported microbiome discrimination and combined AUCs came from the animal component, not from a validated human retrieval model.

A prospective cohort published on 22 August 2026 analyzed fecal samples from 25 men with NOA and 14 men with obstructive azoospermia [45]. Within the NOA group, the Firmicutes-to-Bacteroidota ratio yielded an AUC of 0.76 for retrieval. The result was exploratory and lacked independent validation.

### 3.4. Genetic and Genomic Contributions

Genetic information contributes in two distinct ways. Established karyotype and AZF testing form part of routine etiologic assessment. A correctly delimited complete AZFa or AZFb deletion provides established adverse retrieval information when testing follows dedicated guidance [6]. Discovery-scale sequencing instead seeks additional diagnoses and gene-specific prognostic information in selected men with otherwise idiopathic NOA.

In a single-center cohort of 96 North African men negative for routine genetic tests, Kherraf et al. identified likely causal variants in 22 participants [46]. All 12 carriers with variants in meiotic genes had negative micro-TESE. This finding supports a gene- and pathway-specific hypothesis. The ancestry-enriched cohort, sparse gene-level counts, variable expressivity, and absence of independent evaluation preclude a transportable prediction rule.

Riera-Escamilla et al. studied 571 men from three analytic cohorts after excluding known karyotypic and AZF causes [47]. The panel yielded a diagnosis in 6.1%, and likely pathogenic or pathogenic variant carriage was associated with negative TESE. Combined cohort and literature data supported gene-specific counseling, particularly for *TEX11*, *SYCE1* and *MSH4*. Most gene-level counts remained below ten. This was genotype–outcome mapping rather than an externally validated, calibrated patient-level model.

Sequencing studies therefore support etiologic diagnosis and qualified genotype-specific counseling. They do not yet provide externally validated genomic prediction for unselected NOA. Future models must address ancestry, penetrance, variant-classification uncertainty, pipeline transfer, calibration, and incremental value beyond a baseline that already includes karyotype and AZF testing.

### 3.5. Validation Maturity in the Selected Reports

Table 1 presents illustrative reports selected to show different evaluation designs and methodological limitations. The selected reports mainly described discovery, development, or same-source evaluation. They did not document the complete sequence of a prespecified high-dimensional assay–model system, independent-center evaluation, and prospective clinical-utility assessment. Because report selection was purposive, this observation is not a field-wide absence finding.

Evaluation design is more informative than a reported AUC alone. High AUC values in the selected examples mostly arose from case–control, development, or internal-holdout analyses. AUCs from different populations, specimens, procedures, and outcomes are not directly comparable. Table 1 should not support cross-study rankings or prevalence estimates.

## 4. Methodological Barriers to Clinical Evaluation

Clinical evaluation depends on several related requirements. A biologically valid signal may remain unsuitable for clinical use when population definition, assay reproducibility, model development, or external evaluation is inadequate. Performance measured at a later stage does not correct an earlier mismatch between the intended use and the developed assay–model system.

### 4.1. Population, Intended Use and Outcome

NOA includes distinct etiologies and histological patterns, and the prevalence of focal spermatogenesis varies with referral pathways and surgical expertise. Mixing obstructive azoospermia with NOA can produce an easier classification problem that does not represent the target clinic. Restricting the cohort to a narrow idiopathic subgroup may improve internal separation but reduce applicability to genetically defined or treatment-exposed patients. Development and evaluation reports should therefore state eligibility, prior retrieval attempts, etiology, relevant genetic findings, center, time period and reasons for exclusion.

The outcome requires equally precise specification. Patient-level recovery of sperm usable for ICSI is not equivalent to spermatid presence, a histological category, unilateral sampling success, fertilization, pregnancy, or live birth. FNA failure, conventional TESE failure, salvage micro-TESE, and first-attempt micro-TESE are distinct targets. Their baseline risks and clinical meanings differ.

### 4.2. Specimen and Analytical Validity

For seminal-plasma and EV signatures, analytical reproducibility depends on preanalytical and laboratory procedures. Relevant factors include abstinence interval, liquefaction, centrifugation, cellular contamination, storage temperature, freeze–thaw cycles, extraction method, endogenous controls, batch correction, and assay platform. Biospecimen Reporting for Improved Study Quality (BRISQ) addresses biospecimen reporting, and the Minimum Information for Publication of Quantitative Real-Time PCR Experiments (MIQE) addresses quantitative polymerase chain reaction studies [48,49]. The Minimal Information for Studies of Extracellular Vesicles (MISEV2023) covers EV studies [50]. The Minimum Information About a Proteomics Experiment (MIAPE) covers proteomics, and Metabolomics Standards Initiative guidance covers metabolomics [51,52]. Testicular-tissue signatures add spatial sampling heterogeneity and require an invasive specimen when tissue is obtained during retrieval.

A translational study must specify the complete analytical pipeline before clinical evaluation. Required elements include collection instructions, allowable processing delay, specimen fraction, storage, extraction, quality-control material, platform, normalization, assay-failure rules, batch strategy, and cutoff definition. Multi-omics integration adds further alignment and multiplicity requirements.

### 4.3. High-Dimensional Model Development

Omics datasets often contain far more candidate features than retrieval events. Univariate filtering followed by modeling in the same data, normalization using the full dataset, batch correction before data partitioning or feature selection performed outside resampling can leak outcome information and inflate apparent performance. Every data-driven step—including preprocessing, imputation, filtering, tuning and selection—must occur within each resampling loop. Nested cross-validation is needed when hyperparameters are tuned, while bootstrap validation is often more efficient than a single random split for small datasets. The risk is empirical rather than theoretical: selection bias has been demonstrated in high-dimensional gene-expression classifiers when gene selection was not repeated inside every cross-validation iteration [53].

Sample-size justification should reflect the number of outcome events, candidate parameters, anticipated model performance, shrinkage and precision rather than a simplistic events-per-variable rule [54]. Penalization does not remove the need for adequate data, and testing many algorithms does not create independent evidence. A complete model—including intercept, coefficients or executable code, preprocessing, units and thresholds—must be reported so that the model can be specified before evaluation. Average optimism correction is also insufficient when individual predictions are unstable. Repeating the complete development strategy across bootstrap samples can reveal whether selected features, predicted probabilities and implied decisions change materially with sampling variation [55].

### 4.4. Calibration and Transportability

Discrimination describes ranking, not whether predicted probabilities are numerically trustworthy. Counseling requires calibration-in-the-large, calibration slope and a calibration plot in the intended population. A model may preserve a respectable AUC while systematically overestimating or underestimating retrieval probability after transfer to a new case mix or assay platform [56]. External-evaluation sample size should be planned for sufficiently precise estimation of calibration and discrimination, rather than chosen only by convenience [57].

Same-source holdout evaluation tests performance within a local workflow, temporal evaluation tests stability in later patients from the same center, and center-held-out evaluation tests geographic transportability. Multicenter recruitment alone does not constitute geographic validation when participants from all centers are pooled before a random split. Re-selecting features, re-estimating coefficients or re-tuning an algorithm in the evaluation cohort creates a model update and should be reported separately from performance of the original prespecified model. These distinctions and the requirement to evaluate a fully specified model are central to contemporary external-validation guidance [58,59].

### 4.5. Incremental Value, Thresholds and Cost

Clinical utility requires a specified action. A new signature should be compared with a prespecified model containing routine information available at the same decision time, first for calibration and discrimination and then for net benefit at clinically credible thresholds [60]. Decision-curve analysis can support this step, but comparison only with treat-all and treat-none strategies does not demonstrate added value over current clinical information [61]. The clinically relevant contrast is baseline versus baseline plus the molecular signature, evaluated in the same patients, with uncertainty reported for the incremental effect.

Action thresholds should be elicited from patients and clinicians and linked to counseling, referral or procedure planning, not chosen after the fact to optimize sensitivity and specificity. In a small Canadian survey of 22 patients and 17 urologists, patients generally required larger gains in fertility outcomes than clinicians assumed, which is a caution against thresholds set by clinicians alone [62]. Economic evaluation must consider the complete assay cost, failure rate and turnaround time alongside surgical morbidity, delay and the opportunity consequences of a false-negative result. High discrimination alone cannot establish cost-effectiveness. The final evidentiary step is a prospective impact study measuring decisions, benefit, harm, equity and patient-relevant outcomes.

## 5. Routine-Variable Models Define the Comparator Standard

Routine clinical, hormonal, and targeted genetic models currently provide the clearest temporal and geographic evaluations in NOA. They do not establish clinical readiness. They do provide a practical comparator and show how performance can change when case mix, procedure, or setting changes.

Cissen et al. developed a clinical, hormonal, and genetic model in 918 men and evaluated it in 453 men at another fertility center [63]. AUC decreased from 0.69 to 0.65, and validation calibration was described as moderate. This discrimination may be insufficient for clinical use. The design evaluates the model at a center where it was not developed. It also keeps development and external performance distinct.

Ma et al. developed an FNA sperm-retrieval-failure model in 317 men [64]. Prospective evaluation included 61 later patients at the same center and 219 patients at another center. The development AUC was 0.823, with a bootstrap-corrected value of 0.816. Validation was reported mainly through classification accuracies of 85.25% and 83.56%, not external AUC and calibration. Bachelot et al. trained 8 algorithms in 175 retrospective patients and evaluated them prospectively in 26 later patients [65]. The selected random forest had an AUC of 0.90. The small same-center temporal cohort did not establish geographic transportability.

Zheng et al. developed a model in 261 men with idiopathic NOA and evaluated it prospectively in 48 later same-center patients [66]. Development AUC was 0.811, and temporal-cohort accuracy was 85.42%. Russo et al. used data from 333 men across six centers [67]. The internally bootstrap-corrected concordance index was 0.75. Multicenter recruitment and bootstrap correction strengthen development, but neither replaces evaluation of a prespecified model at a held-out center. The Russo model also incorporated histology and was therefore procedure-informed.

Jiang et al. later evaluated the existing Ma model in 769 FNA patients and fitted reconstructed models in that same cohort [68]. The newly fitted models had AUCs of 0.865 (95% CI 0.838–0.892) and 0.876 (95% CI 0.850–0.921). These estimates do not describe external performance of the inherited model. A separate 140-patient micro-TESE cohort served a procedure-comparison and triage purpose; it was not an external validation dataset for the reconstructed models. This example shows why reports, models and evaluations cannot be counted interchangeably and why transport across retrieval procedures requires a new target definition.

Wang et al. developed a tissue-morphometry model in 313 men, with a development AUC of 0.839, and evaluated it in 93 men at another center [69]. The external cohort was summarized by accuracy (89.25%), without an external AUC or calibration assessment. Because the model uses testicular morphometry and the Johnsen score, it is procedure-informed rather than a noninvasive preoperative tool.

In Klinefelter syndrome, Gül et al. used a 12-center internal dataset split into 246 training patients and 61 test patients and an independent-center cohort of 163 men. External random-forest discrimination remained high (AUC 0.953), while accuracy fell to 0.834 and calibration deteriorated [70]. Ranking survived the move between centers; the predicted probabilities did not. That is the argument for reporting calibration and clinically meaningful thresholds alongside AUC, and a model built within one diagnosis should not be carried across to unselected NOA.

Ceyhan et al. retrospectively developed a nomogram in 3093 men with NOA treated across multiple centers [71]. The accessible abstract and publisher materials do not document a prespecified, distinct validation cohort or report calibration performance. The authors also call for further validation. External-validation and calibration status are therefore recorded as not verified from accessible primary-source material, rather than absent.

Xi et al. reported an extreme gradient boosting (XGBoost) model based on routine preoperative variables in more than 2800 men [72]. The official abstract reports a mean AUC of 0.9183 and AUCs of 0.8469 and 0.8301 in the reported internal and external cohorts, respectively. The accessible abstract does not specify the composition or size of those cohorts. It also does not report calibration or decision-analytic performance. This is therefore a reported external-cohort evaluation whose provenance and probability calibration could not be independently verified.

Future omics studies should demonstrate incremental value over a prespecified routine-variable benchmark. The comparison should use the same patients and prediction time. It should preserve the clinical model specification and quantify changes in calibration, discrimination, risk classification, and net benefit. This requirement is more informative than a molecular AUC alone. Table 2 summarizes selected routine-variable prediction reports and their principal methodological lessons for future omics models.

## 6. An Author-Developed Seven-Gate Validation Framework

**Figure 2:**
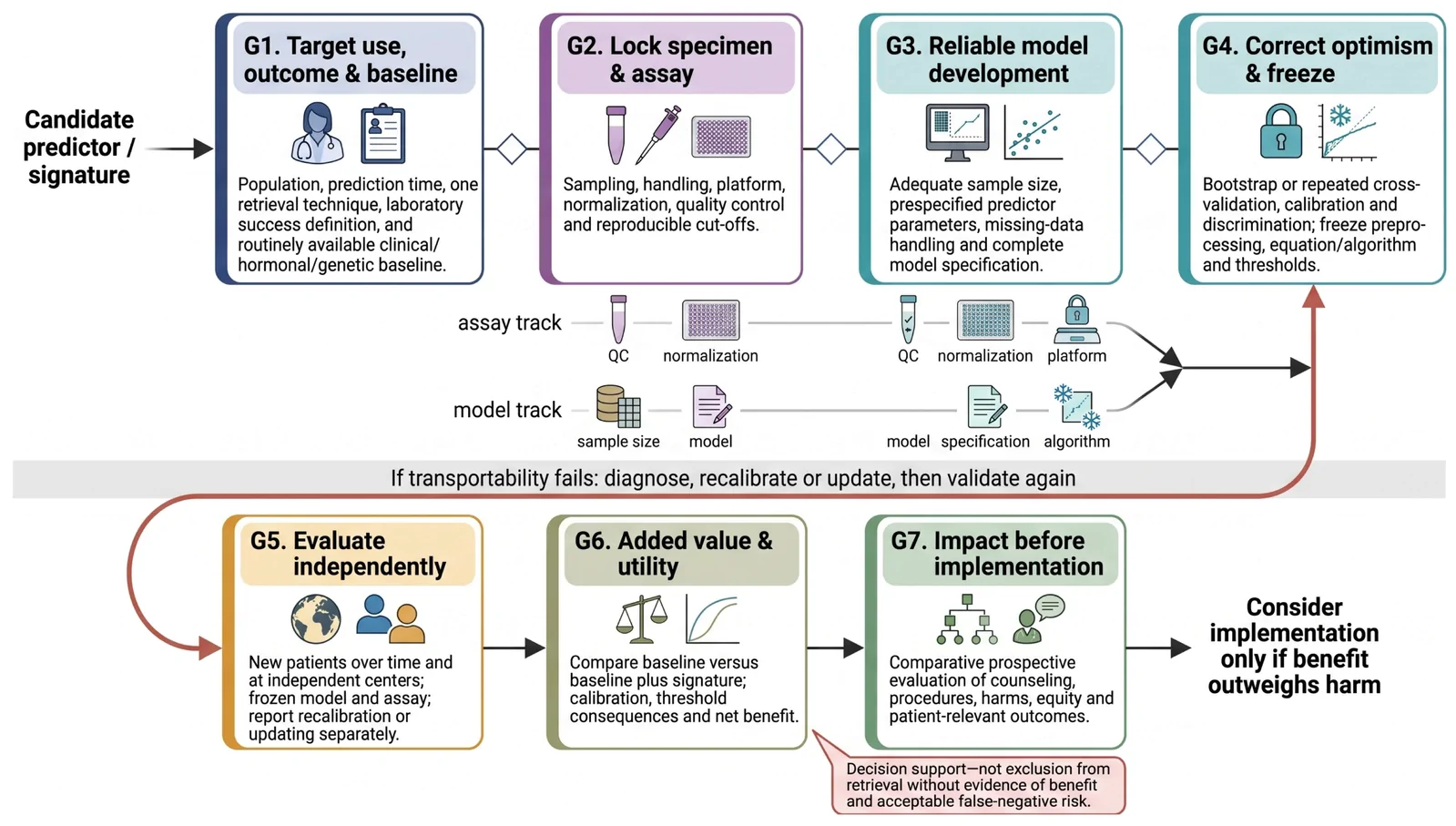
Author-developed seven-gate framework for clinical evaluation of omics-based sperm-retrieval prediction. G1 specifies the population, prediction time, procedure, outcome, and routine-data comparator. G2 specifies specimen handling, assay platform, normalization, quality control, and cutoff. G3 and G4 address model development and internal validation. G5 evaluates the prespecified assay–model system in later patients and independent centers. G6 assesses incremental value, calibration, threshold consequences, and net benefit. G7 evaluates prospective clinical impact. Model updating after inadequate external performance requires further independent evaluation. The framework is not a validated score, consensus statement, or clinical practice guideline. Created in BioRender. Kaltsas, A. (2026) https://BioRender.com/485xl78, accessed on 14 August 2026. Connecting lines and arrows indicate progression through the evaluation framework. Diamonds mark checkpoints between stages. The red curved pathway indicates model recalibration or updating followed by renewed validation when transportability is inadequate.

The proposed framework organizes evidence required for clinical evaluation into seven gates. Gates 1 and 2 define the clinical question and assay. Gates 3 and 4 address model development and internal validity. Gates 5 and 6 address independent evaluation and incremental clinical value. Gate 7 addresses prospective clinical impact. Completion of a later gate does not correct deficiencies at an earlier gate. Figure 2 shows this sequence and the return path after inadequate external performance.

The framework is an author-developed synthesis, not a validated scoring instrument, consensus statement, or clinical practice guideline. Its prediction-model components are informed by CHARMS, TRIPOD, TRIPOD+AI, PROBAST, PROBAST+AI, TRIPOD-SRMA, and external-validation guidance [14,15,16,17,18,19,58,59]. Its assay components draw on BRISQ, MIQE, MISEV2023, MIAPE, and metabolomics reporting standards [48,49,50,51,52]. Its clinical-value components draw on added-value methods, decision-curve analysis, and early clinical-evaluation guidance [60,61,73]. Its contribution is the integration and ordering of these established requirements for preoperative sperm-retrieval prediction in NOA.

### 6.1. Define the Clinical Question and Specify the Assay—Gates 1–2

Gate 1 specifies the target population, setting, decision-maker, prediction time, retrieval procedure, laboratory definition of success and action the prediction is intended to inform. A preoperative micro-TESE model should use only information available at counseling and predict patient-level recovery of usable sperm—not histology, etiological class, intraoperative detection or downstream live birth. The routine-variable comparator must be prespecified at the same decision time.

Gate 2 converts the discovery workflow into a reproducible assay. The protocol should prespecify specimen collection, processing delay, fraction, storage, extraction, platform, normalization, quality controls, analytical precision, assay-failure rules, and cutoffs. Inter-laboratory reproducibility must be measured before cross-center transportability is claimed. A model cannot compensate for unstable laboratory input.

### 6.2. Develop the Model and Assess Internal Validity—Gates 3–4

Gate 3 requires a justified sample size, prespecified candidate predictors and modeling strategy, appropriate handling of missing data and clustering, and full publication of preprocessing, units, intercept, coefficients or executable code. Every outcome-informed preprocessing and feature-selection step must occur inside resampling.

Gate 4 uses bootstrap validation or appropriately nested repeated cross-validation to quantify optimism in the complete pipeline. Optimism-corrected discrimination, calibration and overall performance should be reported with confidence intervals. A single random split is an inefficient use of a small dataset and provides only internal evaluation, regardless of whether it is labeled a validation set.

### 6.3. Assess External Performance and Incremental Value—Gates 5–6

Gate 5 evaluates an assay and model specified before outcome access. Temporal testing in later patients examines stability. Independent-center evaluation examines geographic and laboratory transportability. Reports should present case mix, assay failures, calibration, discrimination, and threshold-specific performance. If updating is required, the original model should be reported first and the updated version evaluated again.

Gate 6 compares the routine-data model with the same model plus the molecular signature. Added value should be demonstrated through calibration and clinically useful risk separation, followed by net benefit at thresholds linked to plausible actions and patient preferences. The relevant endpoint is not a statistically larger AUC but better, safer decisions at an acceptable cost.

### 6.4. Evaluate Prospective Clinical Impact—Gate 7

Gate 7 evaluates model-informed care prospectively, with reporting guided by early-stage decision-support standards where appropriate [73]. Outcomes should include understanding and decisional conflict, referral and procedure choices, retrieval outcomes, assay failure, delays, costs, inequity and harms from false reassurance or false-negative exclusion. Implementation should be considered only if the benefit outweighs the harm in the intended population.

A practical program would prioritize a small number of biologically justified signatures, harmonize assays across participating laboratories and enroll consecutive men with strictly defined NOA before a prespecified procedure. Center-aware internal validation would support development; later patients and at least one held-out center would provide independent evaluation. The protocol, assay manual, analysis plan and model specification should be time-stamped before outcome access, with patient representatives helping to define thresholds and the acceptable consequences of false-negative advice.

## 7. Limitations

This critical narrative review used focused PubMed/MEDLINE searching and reference checking rather than an exhaustive, multidatabase systematic search. Reports published before 2010, gray literature, conference proceedings, and studies indexed outside PubMed may have been missed. The title scan and selected abstract audit were not independently duplicated. No review protocol was registered before the work began. These features limit reproducibility and increase the possibility of selection, classification, and interpretation error.

Substantial differences in populations, procedures, specimens, assays, and outcomes precluded pooled comparison. Some reports were available only as abstracts or incomplete publisher records. Missing methodological information was recorded as not verified, not as absent from the full publication. Each tabulated AUC followed the hierarchy in Section 2.4. The design categories were descriptive and did not constitute formal risk-of-bias ratings. The tables are illustrative and should not be interpreted as an exhaustive evidence map, cross-study ranking, or prevalence estimate. This review cannot establish the field-wide absence of independently validated omics systems. Its conclusions are limited to the selected reports and evidence available through 31 August 2026.

## 8. Conclusions

Published molecular studies support associations between omics signals and sperm-retrieval outcomes. The selected reports also show that development or same-source discrimination does not establish calibrated performance in another laboratory or patient population. Clinical evaluation must address the complete assay–model system, including specimen handling, preprocessing, model specification, calibration, and intended use.

A routine-variable model involving more than 2800 participants provides a provisional comparator and reported an external-cohort AUC of 0.8301. Its cohort provenance, calibration, and decision-analytic utility still require independent confirmation. New omics models should therefore assess incremental value beyond a prespecified routine-variable model in the same patients. Evaluation should include calibration, discrimination, threshold-specific consequences, net benefit, assay failure, cost, and patient-relevant outcomes.

The seven-gate framework specifies evidence needed for this evaluation but is not a validated guideline or scoring system. High-dimensional signatures without adequate analytical validation, independent evaluation, and prospective impact evidence should remain within research settings. Established testing for complete AZFa or AZFb deletions and qualified genotype-specific findings should continue to inform counseling according to their separate evidence base.

## Figures and Tables

**Table 1:** Illustrative omics reports selected to demonstrate differences in clinical target, evaluation design, reported performance, and methodological limitations.

Report	Modality/Specimen	Target and Design	Evaluation Sample	Reported Performance	Documented Evaluation Design; Principal Limitation
Xie et al. [24]	EV lncRNA/seminal plasma	NOA retrieval; pooled three-center random split	30 development; 66 holdout	AUC 0.986/0.960	Internal random holdout; no center-held-out assay transfer demonstrated.
Zhang et al. [26]	miRNA/seminal plasma	Three-level outcome; blinded same-setting test	56 development; 40 test	Retrieval AUC 0.927	Blinded same-setting test; fertile controls were included in the three-level framework.
Zhang et al. [31]	EV tRF/serum	NOA retrieval case–control development	30 NOA + 12 controls	AUC 0.954/0.921	Development only; small case–control cohort.
Chen et al. [27]	EV piRNA/seminal plasma	Sequencing then same-source evaluation	20 training; 25 validation	AUC 0.82/0.83	Small same-source holdout; no geographic assay transfer demonstrated.
Kyrgiafini et al. [30]	miRNA/testicular tissue	Mixed azoospermia–cryptozoospermia; outcome-defined groups	40 men; 10 pooled libraries	Differential expression; no patient-level model	Biological discovery; surgical specimen, pooled libraries and no patient-level model.
Lv et al. [36]	circRNA/serum	NOA retrieval model development	180 total	AUC 0.977	Model development; no clearly separate evaluation cohort verified.
Shi et al. [37]	circ_MGLL/testicular tissue	Idiopathic NOA; single-center split	58 development; 56 test	AUC 0.868/0.811	Same-center internal split; invasive, procedure-informed predictors.
Babakhanzadeh et al. [32,33,34,35]	circRNA ceRNA axes/plasma	Successful versus failed retrieval within NOA; single-center case–control	60 NOA (29 retrieval-positive, 31 retrieval-negative) + 40 fertile controls; apparently the same cohort in all three reports	AUC 0.920; 0.983; 0.956 and 0.970; two additional axes reported above 0.90	Case–control development in an apparently overlapping cohort; the 2026 post-cutoff abstract did not report sample size or evaluation design.
Korbakis et al. [38]	TEX101/seminal plasma	NOA subset; spermatozoa-or-spermatid endpoint	26 NOA	AUC 0.69 (95% CI 0.48–0.89)	Assay/predictor evaluation in a small retrieval subset; imprecise estimate.
Fietz et al. [40]	Proteomics/seminal plasma	Phenotype-group discovery	7–8 per group	Candidate HSPA2 and LDHC	Discovery; no prespecified patient-level prediction model.
Gilany et al. [41]	GC–MS metabolomics/seminal plasma	TESE−/TESE+/fertile controls	11/9/10	ROC areas > 0.88	Exploratory case–control development; no independent evaluation.
Cheng et al. [42]	Metabolomics/seminal plasma	Cryptorchidism-associated NOA	35 cryptorchidism-associated azoospermia + 40 controls overall; retrieval subgroup *n* = 21 (11 SR+, 10 SR−)	Differential metabolic profile	Etiology-specific discovery in a 21-man retrieval subgroup; no external evaluation.
Huang et al. [45]	Gut microbiome/feces	Prospective OA–NOA cohort; retrieval analysis within NOA	39 total; 25 NOA and 14 OA	Firmicutes-to-Bacteroidota ratio AUC 0.76	Exploratory small cohort; no independent evaluation and substantial microbiome-pipeline transfer requirements.
Kherraf et al. [46]	Candidate exome analysis/blood	NOA; genotype–TESE association	96 men; 22 likely causal diagnoses; 12 meiotic-gene carriers	All 12 meiotic-gene carriers had negative TESE	Single-center ancestry-enriched cohort; sparse gene-level counts, no calibrated patient-level model, and no independent evaluation.
Riera-Escamilla et al. [47]	Targeted NGS virtual panel/blood	Idiopathic NOA; three cohorts; genotype–TESE mapping	571 plus published cases	6.1% diagnostic yield	Genotype–outcome mapping; few carriers per gene and no calibrated patient-level model.

AUC, area under the receiver-operating-characteristic curve; ceRNA, competing endogenous RNA; CI, confidence interval; circRNA, circular RNA; EV, extracellular vesicle; GC–MS, gas chromatography–mass spectrometry; lncRNA, long noncoding RNA; miRNA, microRNA; NGS, next-generation sequencing; NOA, non-obstructive azoospermia; OA, obstructive azoospermia; piRNA, PIWI-interacting RNA; SR+, successful sperm retrieval; SR−, failed sperm retrieval; TESE, testicular sperm extraction; tRF, transfer RNA-derived fragment; circ_MGLL, circular RNA derived from the monoacylglycerol lipase gene; HSPA2, heat shock protein family A (Hsp70) member 2; LDHC, lactate dehydrogenase C; n, sample size; ROC, receiver operating characteristic; TEX101, testis-expressed protein 101; TESE+, successful testicular sperm extraction; TESE−, failed testicular sperm extraction. Table 1 is not exhaustive and does not provide prevalence estimates or formal risk-of-bias ratings.

**Table 2:** Selected routine-variable prediction reports: evaluation designs and methodological lessons for omics.

Report	Evaluation Design	Development/Evaluation Sample	Performance Tied to Dataset	Methodological Lesson
Cissen et al. [63]	Independent-center geographic	918/453	AUC 0.69/0.65; validation calibration described	Genuine transportability test; modest discrimination
Ma et al. [64]	Temporal + independent-center	317/61 + 219	Development AUC 0.823; corrected 0.816; validation accuracy 85.25%/83.56%	Keep development and validation metrics distinct
Bachelot et al. [65]	Same-center temporal	175/26	Temporal RF AUC 0.90	Useful intermediate evidence; small cohort and no geographic transfer
Zheng et al. [66]	Same-center prospective temporal	261/48	Development AUC 0.811; temporal accuracy 85.42%	Temporal stability does not establish geographic transportability
Russo et al. [67]	Multicenter development; bootstrap	333 across six centers	Internally corrected C-index 0.75	Multicenter sampling is not held-out-center validation; histology-informed
Jiang et al. [68]	Existing-model evaluation + updating	Existing model in 769; separate micro-TESE comparator *n* = 140	Reconstructed-model AUCs 0.865/0.876 in fitting cohort	Updating is not external validation; procedure shift changes the target
Wang et al. [69]	Independent-center geographic	313/93	Development AUC 0.839; external accuracy 89.25%	Procedure-informed; external AUC and calibration unavailable
Gül et al. [70]	Independent-center geographic	246 training + 61 internal/163 external	External RF AUC 0.953; accuracy 0.834; calibration worsened	High AUC can coexist with clinically important calibration drift
Ceyhan et al. [71]	Retrospective multicenter development; validation status not verified	3093 men	SRR 50.2%; age and testicular volume retained in multivariable analysis	Large multicenter development does not itself establish evaluation in a distinct prespecified cohort
Xi et al. [72]	Reported internal and external cohorts; exact provenance not independently reconstructed	>2800 overall	Mean AUC 0.9183; internal 0.8469; external 0.8301	Provisional routine-variable comparator; cohort composition, calibration, and clinical impact require independent confirmation

AUC, area under the receiver-operating-characteristic curve; C-index, concordance index; micro-TESE, microdissection testicular sperm extraction; RF, random forest; SRR, sperm-retrieval rate; n, sample size. The table is illustrative, not exhaustive, and does not imply that the listed models are ready for routine use.

## Data Availability

No new participant-level data were generated or analyzed for this narrative review. The reproducible PubMed strategy, selection rules, revision audit, and source-access notes are provided in Supplementary File S1.

## Supplementary Materials

The following supporting information can be downloaded at: https://www.mdpi.com/article/10.3390/genes17091088/s1, Table S1: Search platform, dates, yield, restrictions, and narrative-screening record; Table S2: Potentially relevant records assessed during revision and the principal table decision; Table S3: Access status for reports central to revised claims. References [35,45,71,72,74,75,76,77,78,79,80,81,82,83,84,85,86,87,88,89,90,91] are cited in the Supplementary Materials.

## Abbreviations

The following abbreviations are used in this manuscript:

AUC	Area under the receiver-operating-characteristic curve
AZF	Azoospermia factor
BRISQ	Biospecimen Reporting for Improved Study Quality
CHARMS	CHecklist for critical Appraisal and data extraction for systematic Reviews of prediction Modeling Studies
ceRNA	Competing endogenous RNA
CI	Confidence interval
circRNA	Circular RNA
ELISA	Enzyme-linked immunosorbent assay
EV	Extracellular vesicle
FNA	Fine-needle aspiration
FSH	Follicle-stimulating hormone
GC–MS	Gas chromatography–mass spectrometry
ICSI	Intracytoplasmic sperm injection
LASSO	Least absolute shrinkage and selection operator
LC–MS/MS	Liquid chromatography–tandem mass spectrometry
lncRNA	Long noncoding RNA
MIAPE	Minimum Information About a Proteomics Experiment
micro-TESE	Microdissection testicular sperm extraction
MIQE	Minimum Information for Publication of Quantitative Real-Time PCR Experiments
miRNA	MicroRNA
MISEV2023	Minimal Information for Studies of Extracellular Vesicles (2023)
mRNA	Messenger RNA
NGS	Next-generation sequencing
NOA	Non-obstructive azoospermia
OA	Obstructive azoospermia
PCA	Principal component analysis
piRNA	PIWI-interacting RNA
PROBAST	Prediction model Risk Of Bias ASsessment Tool
PROBAST+AI	PROBAST extension for artificial intelligence
qPCR	Quantitative polymerase chain reaction
QC	Quality control
ROC	Receiver-operating characteristic
SANRA	Scale for the Assessment of Narrative Review Articles
SRR	Sperm-retrieval rate
TESA	Testicular sperm aspiration
TESE	Testicular sperm extraction
tRF	tRNA-derived fragment
TRIPOD	Transparent Reporting of a multivariable prediction model for Individual Prognosis Or Diagnosis
TRIPOD+AI	TRIPOD extension for artificial intelligence
TRIPOD-SRMA	TRIPOD extension for systematic reviews and meta-analyses
XGBoost	Extreme gradient boosting
