# Supplementary material for: Omics-Based Sperm-Retrieval Prediction in Non-Obstructive Azoospermia: A Critical Narrative Review and Validation Framework
Source: Genes (Basel). 2026 Sep 10;17(9):1088. doi: 10.3390/genes17091088 (PMC13606654; doi:10.3390/genes17091088)
Supplement: Supplementary file 1 [file genes-17-01088-s001.zip › genes-4543341-supplementary.pdf]

SUPPLEMENTARY FILE S1

Search Strategy and Evidence-Selection Audit

Omics-based sperm-retrieval prediction in non-obstructive azoospermia

Search updated 31 August 2026

Reference numbers in square brackets correspond to the reference list of the main article.

S1. Purpose and review design

This supplement documents the focused PubMed/MEDLINE mapping exercise that informed the revised critical narrative review. It supports transparency and reproducibility without converting the article into a systematic review. The search and audit do not support PRISMA flow counts, pooled estimates, formal duplicated risk-of-bias assessment, or a prevalence estimate of methodological limitations.

S2. Search record

Table S1. Search platform, dates, yield, restrictions, and narrative-screening record.

Field	Recorded value
Database and platform	PubMed/MEDLINE through the official NCBI E-utilities interface.
Search run	31 August 2026, Europe/Athens.
Publication window	1 January 2010 through 31 August 2026.
Yield	221 unique PubMed records before narrative eligibility assessment.
Query-stage filters	No language, human-study, or document-type filter.

Field	Recorded value
Title scan	Titles of all 221 records were inspected once.
Focused abstract audit	Twenty-two reports received abstract-level audit during revision: directly molecular or potentially omitted records identified in the title scan, together with tabulated reports whose accessible sources required re-checking. Eighteen are recorded in Table S2, two were added to the manuscript and are described in Section S5, and the Ceyhan and Xi reports were re-audited for source access and are recorded in Table S3.
Reference checking	Backward checking of relevant primary reports, reviews, clinical guidance, and methodological publications.
Review process	Single-reviewer narrative mapping; no duplicated systematic screening or protocol registration.
Main Table 1 after revision	Fifteen rows representing 18 molecular reports, because four Babakhanzadeh reports are grouped as an overlapping series.
Main Table 2 after revision	Ten routine-variable reports selected as methodological comparators.

Exact PubMed query

```
(("non-obstructive azoospermia"[tiab] OR "nonobstructive azoospermia"[tiab] OR
"secretory azoospermia"[tiab] OR NOA[tiab]) AND ("sperm retrieval"[tiab] OR "testicular
sperm retrieval"[tiab] OR "testicular sperm extraction"[tiab] OR "microdissection
testicular sperm extraction"[tiab] OR micro-TESE[tiab] OR microTESE[tiab] OR TESE[tiab]
OR "testicular spermatozoa"[tiab] OR "intratesticular sperm"[tiab]) AND (omic*[tiab] OR
genomic*[tiab] OR transcriptom*[tiab] OR proteom*[tiab] OR metabolom*[tiab] OR
lipidom*[tiab] OR epigenom*[tiab] OR microbiom*[tiab] OR microRNA*[tiab] OR miRNA*[tiab]
OR "non-coding RNA*"[tiab] OR "noncoding RNA*"[tiab] OR lncRNA*[tiab] OR circRNA*[tiab]
OR piRNA*[tiab] OR "tRNA-derived fragment*"[tiab] OR exosome*[tiab] OR "extracellular
vesicle*"[tiab] OR ((biomarker*[tiab] OR "molecular marker*"[tiab] OR signature*[tiab]
OR protein*[tiab] OR metabolite*[tiab] OR gene*[tiab] OR genetic*[tiab]) AND
(predict*[tiab] OR prognos*[tiab] OR model*[tiab] OR nomogram*[tiab] OR
outcome*[tiab])))) AND ("2010/01/01"[Date - Publication] : "2026/08/31"[Date -
Publication])
```

The same query with an end date of 13 August 2026 returned 219 records. Two records published on 20 and 22 August 2026 account for the updated total. PubMed counts can change with indexing corrections and should be reported with the run date.

S3. Eligibility and table-selection rules

- Human reports that linked a molecular measurement to patient-level sperm-retrieval outcomes in men with NOA received priority.
- The main molecular table was purposive. Reports were selected to illustrate distinct modalities, intended uses, evaluation designs, and methodological limitations.
- Mixed obstructive and non-obstructive cohorts, NOA-versus-control classification, histology prediction, intraoperative detection, and post-retrieval outcomes were retained only as methodological context unless a separable retrieval analysis was available.
- First-attempt and salvage procedures were treated as distinct targets. FNA, TESA, conventional TESE, and micro-TESE were also distinguished.
- Multiple reports with apparently overlapping cohorts were grouped and were not counted as independent replication.
- Conference abstracts and inaccessible full texts were not used to infer that unreported methods were absent. Only explicitly reported information was extracted, and unresolved items were labeled not verified.
- Routine-variable reports were selected for Table 2 when they illustrated temporal, geographic, calibration, updating, or incremental-value issues relevant to future omics models.

S4. Potentially relevant records not included in the main tables

Table S2 is a revision audit, not a complete exclusion log for all 221 records. The table records direct or adjacent reports identified during title and selected abstract review and gives one principal reason for non-tabulation. A report omitted from the main tables is not judged unimportant.

Table S2. Potentially relevant records assessed during revision and the principal table decision.

Record	Decision	Principal reason
Abdallah et al. [74], 2026. Seminal long non-coding RNAs as prognostic non-invasive biomarkers in NOA. PMID 41530831.	Context; not tabulated	Direct candidate panel, but the abstract does not provide a clearly separated evaluation cohort or complete TESE subgroup reporting.
Larriba et al. [75], 2024. Seminal EV sncRNA sequencing and miRNA/isomiR profiles. PMID 37245055.	Context; not tabulated	Direct and relevant, but the main table already contains EV-RNA examples and the study remains a discovery plus same-source verification design.
Ghanami Gashti et al. [76], 2023. PGK2 and ACR proteins in seminal plasma.	Context; not tabulated	Targeted two-protein study with a small same-source cohort; not a high-dimensional assay–model evaluation.

Record	Decision	Principal reason
PMID 35930251.		
Cao et al. [77], 2023. Seminal plasma ExLncRNA pairs using WGCNA. PMID 37394828.	Context; not tabulated	Direct retrieval biomarker analysis; same-source model development and possible cohort overlap require further verification.
Gamidov et al. [78], 2022. Seminal plasma lipidomics and micro-TESE outcome. PMID 36098587.	Context; not tabulated	Direct lipidomic report; Russian-language full text was not assessed, and the accessible abstract describes a small development analysis.
Ibis et al. [79], 2022. PCNA and LIM15 expression and sperm retrieval. PMID 35570072.	Context; not tabulated	Prospective candidate-gene study, but it evaluates two targeted markers rather than a high-dimensional omics system.
Willems et al. [80], 2023. Semen and urine microRNA biomarkers. PMID 36983773.	Context; not tabulated	The endpoint includes spermatozoa and spermatogonia detection and is not fully equivalent to preoperative recovery of usable sperm.
Amer et al. [81], 2023. Seminal plasma resistin and leptin. PMID 37547577.	Context; not tabulated	Targeted protein markers with subgroup analysis and no independent evaluation.
Hashemi et al. [82], 2020. Testis-specific genes in seminal plasma. PMID 31793700.	Context; not tabulated	Small direct candidate-gene study with limited model validation.
Pansa et al. [83], 2014. ESX1 mRNA in seminal fluid. PMID 25316452.	Context; not tabulated	Direct single-marker study; useful historical context but redundant with later seminal-RNA development examples.
Lv et al. [84], 2020. Testicular hsa_circ_0000116. PMID 32735753.	Adjacent; not tabulated	Invasive testicular-tissue marker and procedure-informed evaluation.
Fang et al. [85], 2019. Testicular microRNA profile comparison. PMID 30423284.	Adjacent; not tabulated	Invasive tissue discovery rather than a prespecified preoperative assay–model system.
Cao et al. [86], 2018. Testicular piRNA profiles. PMID 29460056.	Adjacent; not tabulated	Invasive tissue discovery without external evaluation.
Hashemi et al. [87], 2018. Seven testis-specific markers and SPEM1. PMID 30054974.	Adjacent; not tabulated	Tissue-based targeted marker analysis and small same-source evaluation.
Ando et al. [88], 2012. Testicular VASA	Adjacent; not	Invasive tissue measurement and procedure-informed

Record	Decision	Principal reason
mRNA. PMID 21979299.	tabulated	target.
Stahl et al. [89], 2011. Testicular HSFY mRNA. PMID 21676392.	Adjacent; not tabulated	Invasive tissue measurement rather than a noninvasive preoperative system.
Kleiman et al. [90], 2011. CDY1 and BOULE transcripts in biopsy. PMID 21474125.	Adjacent; not tabulated	Markers were assessed in the same biopsy used for retrieval, so the design is not preoperative transportable prediction.
Gilany et al. [91], 2018. Raman metabolic fingerprinting. PMID 30009145.	Context; not tabulated	Small discovery study; a later Raman report is discussed in the main text.

S5. Post-cutoff records added to the manuscript

Two studies published after the original 13 August 2026 cutoff were added to the revised manuscript. Their abstracts broaden the evidence map but do not establish independent-center assay transfer, calibrated probabilities, or prospective clinical utility.

Record	Evidence reported	Interpretation in revision
Babakhanzadeh et al. [35], 20 August 2026. PMID 42623004; DOI 10.1007/s10815-026-04013-x.	Two circulating ceRNA axes with reported AUCs above 0.90; the abstract does not state sample size or evaluation design.	Added to the RNA section and grouped with the earlier Babakhanzadeh series. Potential cohort overlap remains not verified.
Huang et al. [45], 22 August 2026. PMID 42629513; DOI 10.1007/s10815-026-04002-0.	Prospective cohort of 39 men, including 25 with NOA. The Firmicutes-to-Bacteroidota ratio yielded an exploratory AUC of 0.76.	Added to the microbiome section and Table 1. No independent evaluation was reported.

S6. Source-access audit

Table S3. Access status for reports central to revised claims.

Report	Accessible primary material	Permitted interpretation
Ceyhan et al. [71]	PubMed abstract and official publisher record; full methodological detail not	Multicenter retrospective development in 3,093 men. Distinct validation and calibration

Report	Accessible primary material	Permitted interpretation
	available in the accessed material.	are recorded as not verified, not absent.
Xi et al. [72]	Official publisher abstract; listed supplementary file could not be retrieved during audit.	Mean AUC 0.9183; internal AUC 0.8469; reported external AUC 0.8301. Cohort composition, calibration, and decision analysis remain not verified.
Babakhanzadeh et al. [35]	PubMed publisher abstract for an ahead-of-print article.	Two ceRNA axes with AUCs above 0.90. Sample size, evaluation design, and overlap with earlier reports remain not verified.
Huang et al. [45]	Structured PubMed abstract with cohort sizes and exploratory AUC.	Small prospective association with retrieval; no independent validation reported in the abstract.

S7. Interpretation boundaries

The mapping exercise used one database and title/abstract-focused terms. Records lacking NOA, retrieval, or molecular terminology in the title or abstract may have been missed. Engineering literature indexed outside PubMed and gray literature were not comprehensively searched. The search yield is therefore a reproducible narrative map, not an exhaustive denominator.

No audited abstract clearly documented the complete sequence of a prespecified high-dimensional assay–model system, inter-laboratory transfer, independent-center probability validation, and prospective clinical-utility assessment. This statement is limited to the records identified and examined. It should not be interpreted as proof that no qualifying report exists anywhere in the field.
